# Time- and Space-Varying Atmospheric Phase Correction in Discontinuous Ground-Based Synthetic Aperture Radar Deformation Monitoring

**DOI:** 10.3390/s18113883

**Published:** 2018-11-11

**Authors:** Zengshu Huang, Jinping Sun, Qing Li, Weixian Tan, Pingping Huang, Yaolong Qi

**Affiliations:** 1 Electronics & Information Engineering, Beihang University, Beijing 100191, China; zengshu_huang@163.com (Z.H.); longgniy@163.com (Y.Q.); 2 Department of Engineering, University of Cambridge, Cambridge CB12PZ, UK; qingli@buaa.edu.cn; 3 College of Information Engineering, Inner Mongolia University of Technology, Hohhot 010051, China; wxtan@imut.edu.cn (W.T.); hwangpp@imut.edu.cn (P.H.)

**Keywords:** discontinuous GB-SAR, deformation monitoring, time- and space-varying characteristic, atmospheric phase correction, coherent scatterers

## Abstract

Ground-based synthetic aperture radar (GB-SAR) uses active microwave remote-sensing observation mode to achieve two-dimensional deformation measurement and deformation trend extraction, which shows great prospects in the field of deformation monitoring. However, in the process of GB-SAR deformation monitoring, the disturbances caused by atmospheric effect cannot be neglected, and the atmospheric phases will seriously affect the precision of deformation monitoring. In discontinuous GB-SAR deformation monitoring mode, the atmospheric phases are particularly affected by changes of time and space, so the traditional models of atmospheric phase correction are no longer applicable. In this paper, the interferometric phase signal model considering atmospheric phase is first established. Then, the time- and space-varying characteristics of the atmospheric phase are analyzed, and a novel time- and space-varying atmospheric phase correction algorithm, based on coherent scatterers analysis, is proposed. Finally, slope deformation monitoring experiments are carried out to verify the validity and robustness of the proposed algorithm.

## 1. Introduction

Natural disasters, such as landslides and mudslides, seriously threaten people’s personal and property safety. Ground-based synthetic aperture radar (GB-SAR) can extract the deformation value and the deformation trend of the overall monitoring area, which has become an important technical means for the deformation monitoring of slopes, dams, bridges, etc. [1,2,3,4,5,6,7,8]. Compared with space-borne and airborne SAR, GB-SAR has the advantages of a short time baseline, little spatial baseline error, and flexible observation mode, which make it a powerful supplement to space-borne SAR and airborne SAR in the field of deformation measurement [9,10]. Although the observation distance of GB-SAR is closer than that of space-borne SAR and airborne SAR, the atmospheric disturbances can still bring interferometric phase delay, which will seriously affect the accuracy and reliability of the deformation production [10,11,12,13]. Therefore, the study of the estimation and correction algorithm of the GB-SAR atmospheric phase is important in the process of GB-SAR interferometry.

Atmospheric disturbances, caused by the difference in the atmospheric temperature, pressure, and humidity in the electromagnetic wave propagation, are shown as the delay phases in radar images. The atmospheric delay phase has always been an important factor affecting the development of the InSAR application. Current InSAR atmospheric phase correction algorithms are mainly divided into two categories. One is based on the external auxiliary data, which mainly includes the ground meteorological data, GPS data, and space radiation measurement data [12,13]. However, the accuracy is limited to the sparse degree of the data acquisition station, and high acquisition density will undoubtedly result in additional work burden and cost. The other kind of atmospheric phase correction algorithm is based on the interferometric data [10,11,14,15], which is not limited to external devices and has a strong adaptive capacity. Therefore, the methods based on the interferometric data are the most common solutions. GB-SAR has two different data acquisition modes, named continuous GB-SAR and discontinuous GB-SAR [16]. In continuous GB-SAR deformation monitoring, the system is usually installed in a fixed position and the data acquisition intervals are regular, which is suitable for fast displacement monitoring scenes. In comparison, the discontinuous GB-SAR deformation monitoring has a long acquisition interval, which is a cost-effective approach for slow displacement monitoring scenes [17]. However, atmospheric phases are greatly affected by the changes of time and space in discontinuous GB-SAR, and the temporal decorrelation is serious. If the atmospheric phase correction methods of continuous GB-SAR are still exploited, the atmospheric phase correction will be inaccurate, or even incomplete.

In order to solve the above problem, a novel time- and space-varying atmospheric phase correction algorithm, based on coherent scatterers (CSs) analysis, is proposed. First, a GB-SAR interferometric phase model considering the atmospheric phase is established. Then, the CSs are extracted by the frequency interleaved sub-images entropy method and local spatial consistency analysis. Compared with the existing atmospheric phase correction algorithm, using permanent scatterers analysis methods to extract stable control points (SCPs), the proposed algorithm can complete stable control point extraction through a single GB-SAR pair [18,19], which is more suitable for the discontinuous GB-SAR deformation monitoring. Furthermore, the local spatial correlation of the atmospheric phases is used to acquire SCPs. Then, the estimation of the time- and space-varying atmospheric phase is completed by using the spatial interpolation, based on the Delaunay triangulation and the polynomial fitting in the range and azimuth direction. Compared with the existing atmospheric phase correction algorithm, which needs time series data and does not consider the space-varying characteristics of the atmospheric phases, the algorithm proposed in this paper is more suitable for atmospheric phase correction in discontinuous GB-SAR monitoring.

This paper is organized as follows. In Section 2, the geometric and signal models of GB-SAR deformation monitoring are introduced, and the physical signal model along with the time- and space-varying characteristics of the atmospheric phase are analyzed. In Section 3, based on the local spatial correlation analysis of the CSs, a novel time- and space-varying atmospheric phase correction algorithm for discontinuous GB-SAR deformation monitoring is proposed. In Section 4, slope deformation monitoring experiments are carried out, and the validity of the proposed algorithm is verified by comparing the processing results with those of the linear model method and the total station. Finally, our conclusions are drawn in Section 5. 

## 2. Ground-Based Synthetic Aperture Radar Signal Model and Atmospheric Phase Model

**Figure 1:**
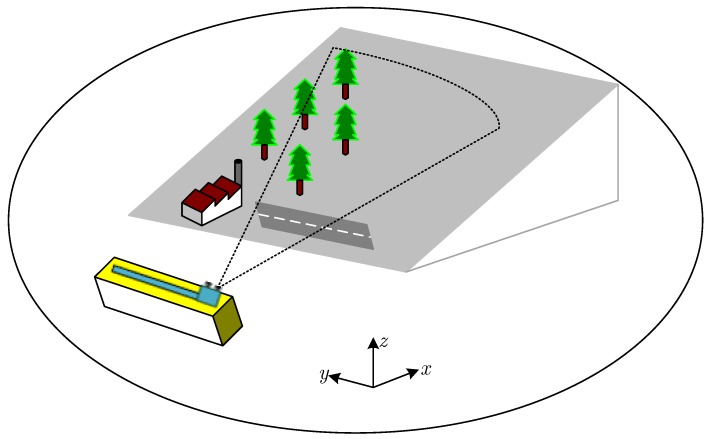
Observation geometry of fround-based synthetic aperture radar (GB-SAR).

The observation geometry of GB-SAR is shown in Figure 1. High-range resolution can be achieved by transmitting and receiving electromagnetic signals in the range direction, and the synthetic aperture is realized through moving along the horizontal rail. Therefore, GB-SAR can realize two-dimensional resolution and zero-baseline interferometry. The signal of GB-SAR after imaging processing can be expressed as

(1) $$S_{i}=\left|\sigma_{i}\right|\times \exp\{-j\frac{4\pi f_{c}}{c}R_{i}\}$$

where *i* is the SAR image series number, $R_{i}=\sqrt{{\left(x_{i}\right)}^{2}+{\left(y-y_{i}\right)}^{2}+H^{2}}$ is the distance from the radar to the target, $\left|\sigma_{i}\right|$ is the target scattering coefficient, *f_c_* is the radar center frequency, and *c* is the speed of electromagnetic wave. For ideal zero-baseline interferometric data, the interferometric processing and deformation retrieval model can be expressed as follows:

(2) $$\{\begin{array}{l} S_{2}S_{1}^{*}=\left|\sigma_{2}\right|\cdot \left|\sigma_{1}\right|\times \exp\{-j\frac{4\pi f_{c}}{c}(R_{2}-R_{1})\}\\ \Delta R=R_{2}-R_{1}=-\frac{\lambda }{4\pi }\Delta \varphi_{in} \end{array}$$

where S* is the conjugate of *S*, ΔR is the displacement between two data acquisition, λ is the signal wavelength, and $\Delta \varphi_{in}$ is the interferometric phase.

However, in GB-SAR field measurement data processing, the interferometric phase includes not only the deformation phase, but also phases such as the atmospheric phase, the noise phase, and the wrapped phase. The GB-SAR interferometric processing is to extract the deformation phase from these phases, and realize the process of deformation retrieval. The interferometric phase model can be expressed as follows:

(3) $$\Delta \varphi_{in}=\varphi_{defo}+\varphi_{atmo}+\varphi_{noise}+\varphi_{rail}+2k\pi$$

where $\varphi_{defo}$ is the deformation phase, $\varphi_{atmo}$ is the atmospheric phase, $\varphi_{noise}$ is the noise phase, $\varphi_{rail}$ is the rail error phase, and *k* is the wrapped phase cycle value. 

The noise phase $\varphi_{noise}$ can be removed by the phase filtering and high-quality pixel selection [20], which can improve the signal-to-noise ratio of the interferometric phases. The noise phase is caused by equipment re-installment in discontinuous GB-SAR; the correction method is presented in [21], and is not the focus of this paper. In order to simplify the analysis, we selected some pairs of images with a long time span to achieve the discontinuous GB-SAR data simulation in this paper, so the rail error phase $\varphi_{rail}$ can be neglected. The wrapped phase cycle value *k* can be solved by the phase-unwrapping algorithm, and the related algorithms are sufficient [22,23,24,25,26]. This paper mainly studies the influence of the atmospheric phase on the GB-SAR interferometric phase and its correction algorithm. Therefore, the interferometric phase model can be simplified as

(4) $$\Delta \varphi_{in}=\varphi_{defo}+\varphi_{atmo}$$

Atmospheric phases of GB-SAR are caused by the difference of atmospheric pressure, temperature, and humidity in different time and space [12]. Therefore, the atmospheric phase model can be deduced by using the atmospheric physical parameters in the deformation monitoring scene. The atmospheric refractive index *N* depends on the atmospheric pressure *P*, the temperature *T*, and the relative humidity *H*. *N* can be expressed as

(5) $$N=N_{dry}+N_{wet}$$

where $N_{dry}$ and $N_{wet}$ are the dry refractive index and the wet refractive index, respectively. The specific expressions are expressed as follows:

(6) $$\{\begin{array}{l} N_{dry}=\frac{77.6\cdot p}{T}\\ N_{wet}=\frac{3.73\cdot 10^{5}\cdot w_{p}}{T^{2}}\\ w_{p}=6.11\cdot e^{\left(19.7\cdot \frac{T-273}{T}\right)}\cdot H \end{array}$$

Therefore, the refractive index *N* is a vector related to the distance vector (*r*) and time (*t*), which can be expressed as follows:

(7) $$N\left(\vec{r},t\right)=N\left(T\left(\vec{r},t\right),P\left(\vec{r},t\right),H\left(\vec{r},t\right)\right)$$

Then, the atmospheric phase can be obtained by integrating on the path *S* of radar electromagnetic wave propagation [12].

(8) $$\varphi_{atm}=10^{-6}\cdot \frac{4\pi }{\lambda }\int_{S}N\left(\vec{r},t\right)dl$$

According to the atmospheric physics model, Noferini built the atmospheric phase linear correction model based on the analysis of the permanent scatterers in [11], which assumes that the atmospheric refractive index was homogeneous in the azimuth direction and that the atmospheric phase had a linear relationship with the observation distance. The linear model is as follows:

(9) $$\varphi_{in}[\vec{r},t,h(\vec{r},t)]=\varphi_{defo}(\vec{r},t)+K\cdot h(\vec{r},t)\cdot r$$

(10) $$\varphi_{atmo}=K\cdot h(\vec{r},t)\cdot r$$

where *K* is an unknown constant and *r* is the distance from radar to observation point. 

The above model parameters can be calculated by using the phase value of the permanent scatterers in the scene, so the GB-SAR time series data is mandatory. The discontinuous GB-SAR has a long acquisition interval, so the dataset is small and the temporal decorrelation is serious. Therefore, the permanent scatterers analysis method is not suitable for atmospheric phase correction in discontinuous GB-SAR deformation monitoring.

## 3. Time- and Space-Varying Atmospheric Phase Correction Algorithm

In this section, a novel time- and space-varying atmospheric phase correction algorithm for discontinuous GB-SAR deformation monitoring is proposed. Discontinuous deformation monitoring is different from that of the continuous mode, and is suitable for the scene with slow deformation and no continuous real-time monitoring. The GB-SAR data in the discontinuous deformation monitoring has the characteristics of little data and a long time baseline, so the frequency interleaved sub-images entropy method is used to extract the CSs as stable control point candidates. Then, according to the local consistency analysis of the atmospheric phases, the candidate points satisfying the requirements are selected as SCPs, which can be used for discontinuous GB-SAR time- and space-varying atmospheric phase correction.

### 3.1. Coherent Scatterers Extraction Using a Single Ground-Based Synthetic Aperture Radar Pair

**Figure 2:**
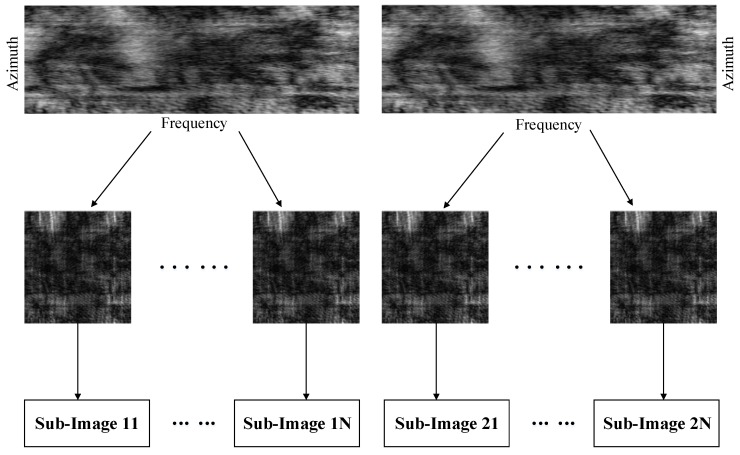
Generation process of frequency interleaved sub-images.

In the continuous deformation monitoring mode, GB-SAR can obtain time series data, so the SCPs can be extracted by using permanent scatterer (PS) methods [11]. For discontinuous deformation monitoring, little data and temporal decorrelation make the PS methods inapplicable. The proposed time- and space-varying atmospheric phase correction algorithm exploits frequency interleaved sub-images entropy method to extract the CSs from a single GB-SAR pair [27]. The GB-SAR system for deformation monitoring in this paper uses the stepped frequency continuous wave signal. When echo data acquisition is completed, the frequency spectrum of GB-SAR data can be achieved. Then the frequency spectrum can be divided into two sub-band spectrums through the frequency interleaved sampling method. The sub-band spectrums, after imaging processing, can be transformed into sub-images [28,29]. The generation process of frequency interleaved sub-images is shown in Figure 2.

Entropy is an alternative approach to estimate coherence in the spectral domain, which can make full use of the sub-image data information by calculating the covariance matrix. The entropy *H* between sub-images is defined by [18]

(11) $$H(i,j)=-\sum_{k=1}^{2N}P_{k}(i,j)\cdot \log_{2}P_{k}(i,j)$$

where (*i*, *j*) is the pixel index, 2*N* is the total number of sub-images, and *P_k_*(*i*, *j*) is

(12) $$P_{k}(i,j)=\frac{\lambda_{k}(i,j)}{\sum_{k=1}^{2N}\lambda_{k}(i,j)}$$

where *λ_k_* represents the eigenvalues of the covariance matrix *C*, and the covariance matrix is given by

(13) $$\left[C\right]=\left[\begin{array}{cccc} E\left\{S_{11}S_{11}^{*}\right\} & E\left\{S_{11}S_{12}^{*}\right\} & \ldots & E\left\{S_{11}S_{2N}^{*}\right\}\\ E\left\{S_{12}S_{11}^{*}\right\} & E\left\{S_{12}S_{12}^{*}\right\} & \ldots & E\left\{S_{12}S_{2N}^{*}\right\}\\ \vdots & \vdots & \ddots & \cdots \\ E\left\{S_{2N}S_{11}^{*}\right\} & E\left\{S_{2N}S_{12}^{*}\right\} & \cdots & E\left\{S_{2N}S_{2N}^{*}\right\} \end{array}\right]$$

where *S* represents the sub-image, and *E*{·} represents the expectation operation.

The entropy value is 0 when the pixels of the sub-images are very similar, and 1 when they are completely unrelated. Therefore, in order to extract the CSs, an appropriate threshold must be set. An adaptive threshold can be obtained by averaging the entropy values of the corner reflectors in the monitoring scene. If the entropy of the pixel is lower than the threshold, the pixel will be selected as a CS. If not, the pixel will be excluded. Some research shows that atmospheric phases have correlation in certain spaces, so in order to prevent a false alarm and to further identify the SCPs, local spatial consistency analysis of the atmospheric phase is performed [15].

### 3.2. Local Spatial Consistency Analysis of the Atmospheric Phase

The local spatial consistency analysis of the atmospheric phase can use the method of StaMPS phase analysis. The StaMPS [30] method mainly uses the spatial correlation of the interferometric phase to evaluate the phase stability of selected pixels. Considering that the atmospheric phases are relevant in local space, the StaMPS analysis method can be used to analyze the local spatial consistency of the atmospheric phases. Firstly, the interferometric phase values of all CSs in a circular region with radius *L* are statistically averaged, which can be expressed as follows:

(14) $$\bar{\phi_{k}}=\sum_{k=1}^{M}\phi_{k}/M$$

where $\phi_{k}$ is the interferometric phase value of the *k*th CS in the circular region, and *M* is the number of CSs in the circular region. If the number of sub-images is *N*, the time correlation coefficient of the *k*th CS in a region with radius *L* can be defined as

(15) $$\gamma_{k}=\frac{1}{N}\left|\sum_{n=1}^{N}\exp\{j(\phi_{k}-\bar{\phi_{k}})\}\right|$$

where $\gamma_{k}\in [0,1]$. The better the local spatial consistency is, the greater its value. Otherwise, the smaller the value is, the worse the local spatial consistency of the CS is. The threshold value $\gamma_{th}$ can be obtained through iterative operation [30]. If $\gamma_{k}\geq \gamma_{th}$, the CS can be considered as a stable control point—otherwise, the CS will be eliminated.

### 3.3. Time- and Space-Varying Atmospheric Phase Estimation and Correction.

**Figure 3:**
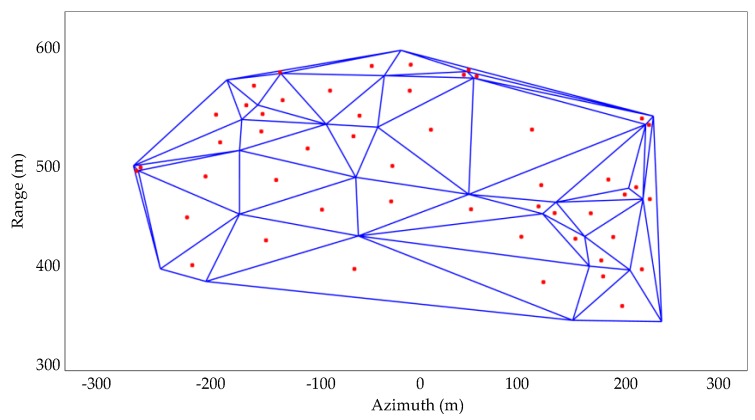
Diagrammatic sketch of Delaunay triangulation. The red points are the pending interpolation points.

After the detection of the SCPs, spatial interpolation is performed to the phase values of the detected SCPs for estimating the atmospheric phase screen (APS) of the whole monitoring scene. The spatial interpolation of a discrete point set is a classical problem in computational geometry. The Delaunay triangulation method is used for the spatial interpolation in this paper. Delaunay triangulation is an optimized spatial structure [31]. There will be no other points in the outer circle of any triangle in the generated triangle net, and the smallest angle of the generated triangle is as large as possible. Therefore, Delaunay triangulation can make the result automatically approach the regular triangle, which is beneficial to improve the interpolation precision. A diagrammatic sketch of Delaunay triangulation is shown in Figure 3.

The realization of Delaunay triangulation first needs the construction of a super triangle [32], which includes all the SCPs and puts their spatial position into the linked list of triangles. The pending interpolation points are inserted in order, and the triangles surrounding the insertion point are extracted from the triangle linked list. Then the common side of the extracted triangles is deleted. Finally, the insertion point and the extracted triangle vertices are connected, and the insertion of a point in the Delaunay triangle linked list is completed. The newly formed triangle can be optimized based on the optimization criterion, and the optimization results are put into the Delaunay triangle linked list. The above steps are performed in loops until all of the pending interpolation points are inserted. The inserted points are interpolated in space according to the cubic method and the phase values of the triangle vertexes where they are located.

However, the SCPs in the monitoring scene often cannot cover the whole scene, so the Delaunay triangulation algorithm can only estimate the atmospheric phases of the regions surrounded by the SCPs, so the APS of the whole scene cannot be obtained. According to Equation (8) in Section 2, the atmospheric phase can be obtained by integrating the refractive index function on the propagation path, so the values of the atmospheric phases change smoothly in the range direction. The relationship between the atmospheric phase and distance can be expressed as follows:

(16) $$\varphi_{atmo}=\sum_{t=0}^{T_{0}}A_{t}R^{t}$$

where *T*_0_ is the fitting order and *A_t_* is the fitting coefficient. For the atmospheric phase values in the range direction, the polynomial fitting is performed after spatial interpolation. The objective function is to minimize the sum of distance squares from the atmospheric phase points $P_{i_{0}}$ to the polynomial curve $\varphi_{atmo}$. The objective function of the polynomial fitting is as follows:

(17) $$F\left(\phi_{atmo}\right)=min\sum_{i_{0}}{\left\|P_{i_{0}}-\phi_{\mathrm{atmo}}\right\|}_{2}{}^{2}$$

After polynomial fitting in the range direction, considering the space-varying characteristics of the atmospheric phases—and that polynomial fitting results also hardly completely cover the entire scene—the polynomial fitting in the azimuth direction is also performed. Then the APS of the monitoring scene is completely obtained, and the atmospheric phase can be corrected according to the interferometric phase model in Equation (4).

(18) $$\varphi_{defo}(\vec{r},t)=\Delta \varphi_{\mathrm{in}}(\vec{r},t)-\varphi_{atmo}(\vec{r},t)$$

**Figure 4:**
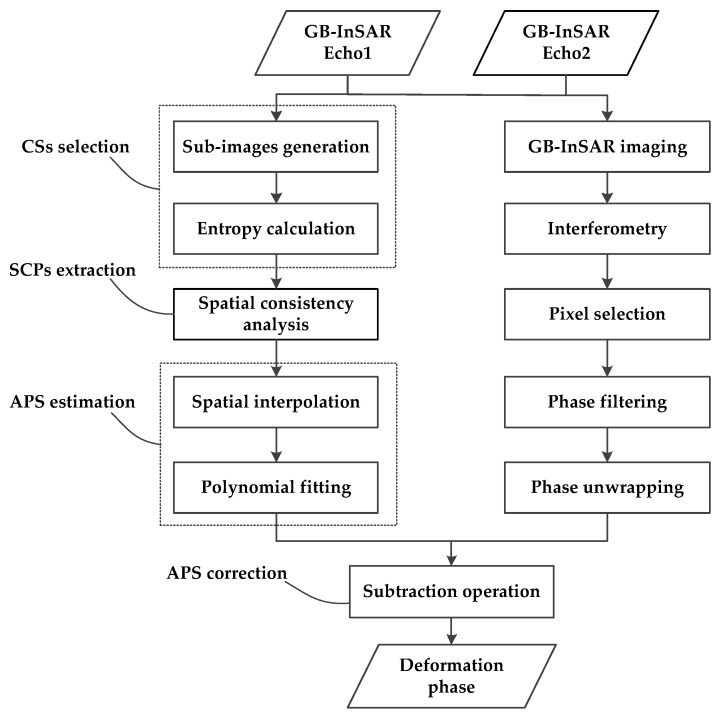
Time- and space-varying atmospheric phase correction algorithm flowchart.

The flowchart of the proposed GB-SAR time- and space-varying atmospheric phase correction algorithm is shown in Figure 4.

## 4. Experimental Results

In order to verify the effectiveness and robustness of the time- and space-varying atmospheric phase correction algorithm proposed in this paper, two slope deformation monitoring experiments in different scenes and atmospheric conditions were carried out. The experimental sites have different climates and height differences. The first experimental site is located in north China, and is a mine park in the suburb of Beijing. The height difference of the observation scene is small and the climate is dry. The second experimental site is located in a mine in Fujian province, in the southern part of China. This scene has large height difference, and the climate in south China is humid. All of the experimental conditions are beneficial for the validation of the proposed time- and space-varying atmospheric phase correction algorithm.

The GB-SAR system used in the experiments is mainly composed of a horizontal rail, a radar transmitting/receiving (T/R) module, and a hardware and software control cabinet. The system uses a stepped frequency continuous wave signal. The maximum observation distance of the system is 5 km. The system parameters are shown in Table 1.

### 4.1. First Case Study: Field Measurements in Northern China

**Figure 5:**
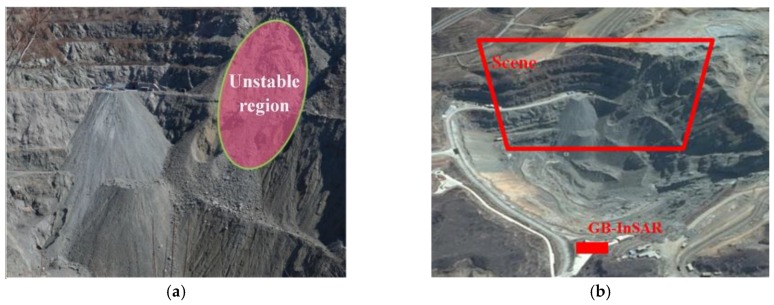
Deformation monitoring for case study 1: (**a**) the optical photo and (**b**) the aerial view on Google Earth.

The first experimental scene is shown in Figure 5. Figure 5a is the optical photo of the monitoring scene, and Figure 5b is the aerial view of the monitoring scene on Google Earth. The right area of the monitoring scene is prone to deformation, which is marked using a red color in Figure 5a. The left area of the monitoring scene is a stable area. The monitoring scene, combining the deformation area and the stable area, is very beneficial to GB-SAR deformation monitoring and data analysis. Two corner reflectors are arranged in the scene, which are used for the calibration and the verification of the later data processing. The arrangement of the corner reflectors is difficult in the deformation area, so all of the corner reflectors are arranged in the stable area.

**Figure 6:**
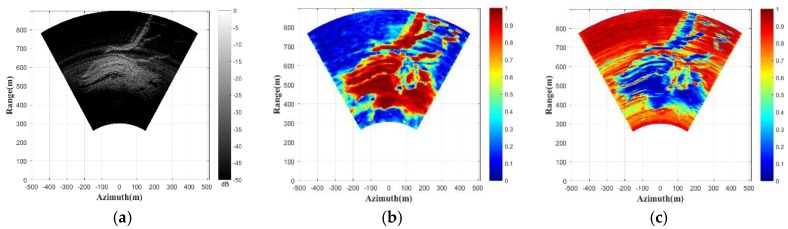

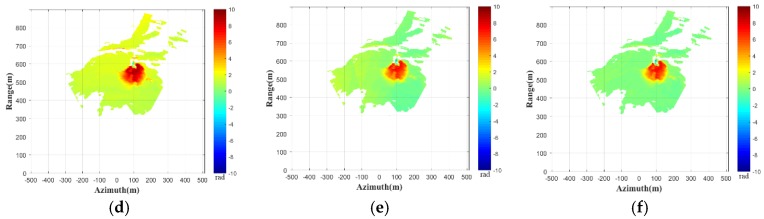
Experimental results: (**a**) reflectivity image; (**b**) correlation coefficient; (**c**) sub-images entropy map; (**d**) uncorrected phases; (**e**) corrected phases using linear model method; and (**f**) corrected phases using proposed method.

The deformation monitoring experiment lasted for one month and the slope was monitored in real time, which was the continuous deformation measurement mode. However, the monitoring scene is a slow deformation scene for the GB-SAR, and the high-frequency data acquisition will increase unnecessary costs. Therefore, the discontinuous deformation monitoring mode is a cost-effective solution. We focus on the discontinuous deformation monitoring data in this case. With respect to the slow deformation scene, the time baseline is increased to achieve the discontinuous deformation monitoring of the scene. A long time baseline will trigger the time-varying characteristics of the environmental parameters. Moreover, the atmospheric refractive indexes are heterogeneous in the azimuth, so the atmospheric phases are space-varying. Therefore, the time- and space-varying atmospheric phase correction algorithm proposed in Section 3 is used for the atmospheric phase correction of the discontinuous deformation monitoring data. Finally, the deformation phases can be obtained. The experimental results are demonstrated in Figure 6.

In the continuous deformation monitoring mode, the data acquisition time of one GB-SAR image is 20 min. In order to obtain the discontinuous deformation data in this experiment, the interferometric data with three-day intervals are selected for processing. Figure 6a shows the reflectivity image of the monitoring scene. The slope area in the monitoring scene has achieved high imaging performance. The correlation coefficient of the GB-SAR data is shown in Figure 6b. The correlation coefficient is a parameter representing correlation of the interferometric pixels [33]. The larger the value of the correlation coefficient, the better the consistency and the phase quality is. Figure 6c is the sub-images entropy map of the GB-SAR interferometric data. The performance of the sub-images entropy is consistent with the correlation coefficient. The pixels in the slope area show good correlation and entropy, and the pixels in the other area are noise, which have poor correlation and entropy. The interferometric phases without atmospheric phase correction are shown in Figure 6d. It can be clearly seen that the atmospheric phases distributed in the scene need to be corrected. The unstable region on the right side of the slope has obvious deformation, which is also consistent with the marked area in Figure 5a. 

**Figure 7:**
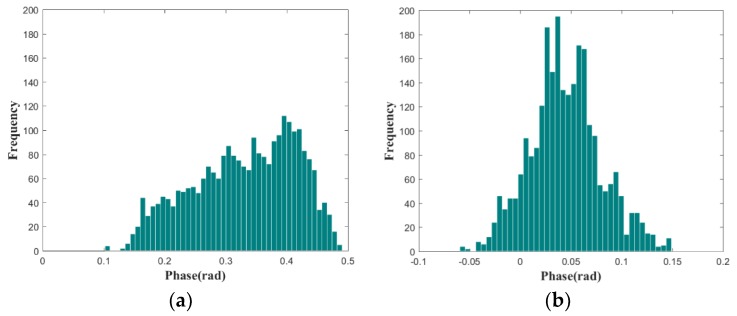
Histograms of the atmospheric phase residuals: (**a**) linear model method and (**b**) our proposed method.

In order to verify the robustness of the proposed algorithm, the atmospheric phase correction algorithm, based on a linear model, is performed for comparison. Figure 6e is the atmospheric phase correction result using the linear model presented in Section 2 [11]. Figure 6f is the result using the atmospheric phase correction algorithm proposed in this paper. It can be qualitatively seen that the correction method based on the linear model has achieved a certain correction effect. However, the atmospheric parameters of the scene in discontinuous GB-SAR deformation monitoring mode are time-varying, and the atmospheric phases have spatial heterogeneity. Therefore, the corrected result using the linear model shows that the atmospheric phases are not corrected completely and the deformation phases on the left side of the slope still have more residual atmospheric phases. The atmospheric phase correction algorithm proposed in this paper extracts CSs through the frequency interleaved sub-images entropy method. Then the local spatial consistency analysis is performed, and the APS can be obtained by the spatial interpolation, based on Delaunay triangulation and the polynomial fitting. Our proposed algorithm not only considers the heterogeneity of the atmospheric phase in azimuth, but also completes the atmospheric phase estimation and correction through detecting enough stable CSs. Therefore, it can achieve better atmospheric phase correction performance than the algorithm using the linear atmospheric phase correction model. A section of the stable area is used for the evaluation of the atmospheric phase residuals. The histograms of the atmospheric phase residual, using the two methods, are shown in Figure 7. The residual phases, using the linear model method as shown in Figure 7a, are mainly distributed in (0.15–0.45 rad). The result of our proposed method, presented in Figure 7b, has smaller residual phases that are mainly distributed in (0–0.1 rad), and the atmospheric phase correction effect is better, which is consistent with our previous conclusion. 

### 4.2. Second Case Study: Field Measurements in Southern China

**Figure 8:**
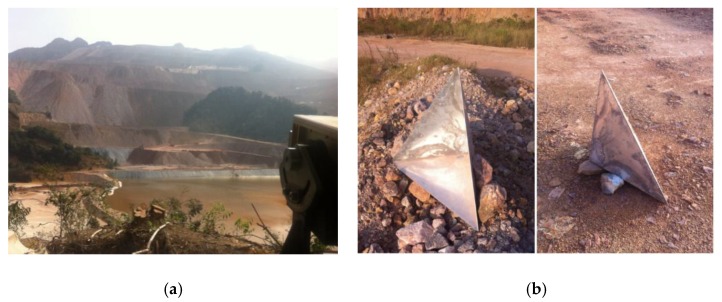
Deformation monitoring for case study 2: (**a**) the monitoring scene and (**b**) corner reflectors.

The monitoring scene of the second case is shown in Figure 8a. The height difference between the monitoring slope and the position of the GB-SAR system is large. The large height difference and the humid climate in southern China make the second monitoring experiment have great significance on the study of atmospheric phase correction. In the first case study, we only conducted the qualitative analysis on the experimental results for the effectiveness validation of the proposed algorithm. In order to further quantitatively analyze the correction accuracy, corner reflectors were set in the monitoring scene, which is shown in Figure 8b. The corner reflectors used in the experiments are large and heavy, and we took strengthening measures to ensure enough stability. The total station instrument was used to measure the deformation data of the corner reflectors, which was essential for the comparison and verification of the GB-SAR results.

**Figure 9:**
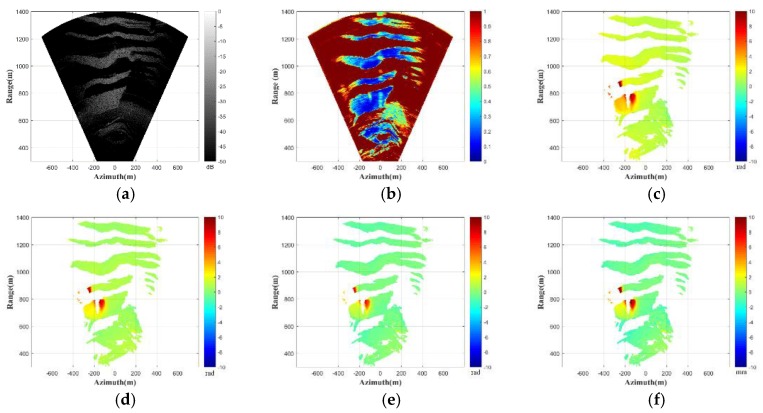
Experimental results: (**a**) reflectivity image; (**b**) sub-images entropy map; (**c**) uncorrected phase; (**d**) corrected phases using the linear model method; (**e**) corrected phase using proposed method; and (**f**) deformation map.

The second experiment lasted 20 days, which realized the real-time deformation monitoring of the slope. The interferometric data with two-day intervals were selected as the discontinuous deformation monitoring data. Due to the great distance from the slope to the location of the GB-SAR system, the closest area is not the region of interest. Therefore, the monitoring range is limited to (400–1400 m) by software settings. The data processing results of the experiment are shown in Figure 9.

Figure 9a shows the radar reflectivity image of the multi-stage slope in scene 2, and it can be seen that the slope stages have achieved good imaging performance. Figure 9b is the obtained frequency interleaved sub-images entropy map, which shows that the entropy values of the slope in the monitoring scene are small. Figure 9c is the interferometric phase before the atmospheric phase correction. It can be seen that serious atmospheric phases are shown in the uncorrected interferometric phase image, which is consistent with the humid climate in southern China and seriously affects the accuracy of deformation monitoring. Figure 9d shows the interferometric phase after the linear atmospheric phase correction, and the correction result is not satisfactory. Figure 9e shows the interferometric phase, using our proposed phase correction method, which achieved good atmospheric phase correction effect. The deformation map corresponding to our corrected phase is shown in Figure 9f. In order to verify the precision of the proposed atmospheric phase correction algorithm, quantitative analysis results are given in Table 2. The deformation results of the corner reflectors in the scene (1) with linear atmospheric phase correction; (2) with our proposed atmospheric phase correction; (3) without the atmospheric phase correction; and (4) the total station deformation measurement results are presented. The observation position of the total station is located at the center of the GB-SAR, and the two corner reflectors are arranged along the radar’s line of sight. As the displacement direction is the radar’s line of sight, the displacement is calculated by projecting the displacement of the total station into the direction of radar’s line of sight. It can be seen that the deformation values of the corner reflectors after our atmospheric phase correction are closer to the measurement results of the total station than the results before correction and of the linear model correction. Actually, the corner reflectors are SCPs, so the theoretical deformation value during the period of the deformation monitoring should be 0, which is consistent with the deformation results after atmospheric phase correction. The above results quantitatively and qualitatively validate that the proposed algorithm can improve the accuracy and reliability of deformation monitoring.

## 5. Conclusions

Atmospheric phase correction plays an important part in GB-SAR deformation monitoring, which is directly related to the precision and reliability of deformation production. In the discontinuous GB-SAR deformation monitoring, the atmospheric phases have time- and space-varying characteristics, and the conventional linear atmospheric phase correction model is no longer applicable. Therefore, in this paper, a novel time- and space-varying atmospheric phase correction algorithm based on CSs analysis is proposed. First of all, the frequency interleaved sub-images entropy method is used to detect the CSs in the scene. Then the extraction of SCPs is completed through the spatial consistency analysis. Finally, the spatial interpolation based on Delaunay triangulation and the polynomial fitting are used to realize the correction of the atmospheric phases. The proposed algorithm can use only a single GB-SAR pair to correct the atmospheric phases in the discontinuous deformation monitoring mode. Two case study results are given for the qualitative and quantitative analysis. Through analyzing the experimental results, and comparing those results with those of the linear atmospheric phase correction model and total station, the validity and reliability of the proposed algorithm are verified.

## Figures and Tables

**Table 1:** System parameters.

Symbol	Parameters	Value
*f_c_*	Center frequency	17 GHz
*B_r_*	Bandwidth	500 MHz
*L_a_*	Rail length	2 m
*N_r_*	Frequency points	10,001
*Na*	Azimuth points	201
*δ_r_*	Range resolution	0.3 m
*δ_a_*	Azimuth resolution	4.3 mrad
*θ_a_*	Antenna beamwidth	60°

**Table 2:** Comparison of results.

Corner Reflectors	Uncorrected Deformation (mm)	Linear Correction (mm)	Our Method Correction(mm)	Total Station (mm)
C_1_	3.0	1.1	0.2	0.5
C_2_	2.6	0.8	−0.1	0.3
